# Risk of dementia among postmenopausal breast cancer survivors treated with aromatase inhibitors versus tamoxifen: a cohort study using primary care data from the UK

**DOI:** 10.1007/s11764-019-00782-w

**Published:** 2019-07-18

**Authors:** Susan E. Bromley, Anthony Matthews, Liam Smeeth, Susannah Stanway, Krishnan Bhaskaran

**Affiliations:** 1 Department of Non-communicable Disease Epidemiology, London School of Hygiene and Tropical Medicine, London, UK; 2 EpiMed Communications Ltd, Abingdon, UK; 3 Royal Marsden NHS Foundation Trust, Sutton, Surrey UK

**Keywords:** Breast cancer, Dementia, Aromatase inhibitors, Clinical epidemiology

## Abstract

**Purpose:**

Among a cohort of postmenopausal breast cancer survivors, we aimed to compare the risk of dementia associated with aromatase inhibitor (AI) therapy versus tamoxifen.

**Methods:**

Using UK primary care electronic health records, we identified 14,214 postmenopausal breast cancer survivors (aged ≥ 54 years) with a first AI or tamoxifen prescription between January 2002 and December 2015 and no previous dementia diagnosis. Women were followed-up to identify incident cases of dementia. Cox regression was used to calculate hazard ratios (HRs) with 95% confidence intervals (CIs) to quantify the association between AI exposure (vs. tamoxifen) and dementia, adjusted for confounders.

**Results:**

A total of 368 incident dementia cases was identified over 57,102 person-years of follow-up. The crude incidence rate of dementia was 7.46 per 1000 person-years (95% CI 6.43–8.65) among women starting endocrine treatment on an AI, and 6.32 per 1000 person-years (95% CI 5.34–7.47) among women starting on tamoxifen. After accounting for age differences and assessing other potential confounders, there was no evidence of a difference in dementia risk between exposure groups (HR for AI vs tamoxifen 1.04, 95% CI 0.83–1.03). There was no evidence of effect modification by age.

**Conclusion:**

There was no evidence for a difference in dementia risk between AI and tamoxifen users among postmenopausal breast cancer survivors.

**Implications for Cancer Survivors:**

Our findings suggest that there is no reason for concern about a difference in dementia risk with AI vs. tamoxifen, which is relevant to postmenopausal breast cancer patients recommended these treatments.

**Electronic supplementary material:**

The online version of this article (10.1007/s11764-019-00782-w) contains supplementary material, which is available to authorized users.

## Introduction

Adjuvant endocrine therapy remains the mainstay of treatment for postmenopausal women with oestrogen-receptor positive breast cancer. Aromatase inhibitors (AIs)—anastrozole, exemestane, and letrozole—have become the preferred treatment in this patient population [1, 2], being superior to tamoxifen in reducing breast cancer recurrence rates and 10-year breast cancer mortality rates [3, 4]. Already widely used drugs, AIs will likely grow in use as the number of breast cancer survivors increases [5], highlighting the importance of evaluating their safety.

Over recent decades, there has been much interest in whether adjuvant endocrine therapy is adversely associated with cognitive functioning in breast cancer survivors—a commonly reported side effect in these patients [6]. Substantial biological evidence supports a role for oestrogen in maintaining cognitive functioning [7], suggesting that the anti-oestrogen effects of endocrine therapy may have detrimental cognitive consequences. Sub-studies of randomized controlled trials (RCTs) have shown that in breast cancer patients, endocrine therapy, either as a group or tamoxifen alone, reduces performance on specific cognitive domains compared with non-cancer controls [8, 9]. Findings from observational studies on this topic have been mixed, with several studies having small sample sizes, a cross-sectional design, or with limited follow-up [7, 10–14].

As the majority of women with oestrogen-receptor positive breast cancer will actually receive treatment with either an AI or tamoxifen, an important question is whether there is a different effect on cognition function between these two treatments. Owing to their different mechanisms of action, circulating oestradiol levels are substantially lower following treatment with AIs than with tamoxifen [15], and could therefore potentially have a greater detrimental effect on cognition. Results from RCT sub-studies [8, 16], however, do not support this hypothesis, with patients on AI performing significantly better on specific cognitive domains than those on tamoxifen. Observational data directly comparing AIs and tamoxifen in this context are limited, with a small meta-analysis of data from six cross-sectional studies reporting no differences between treatments on cognitive performance [11]. There have been no population-based studies directly comparing the effect of AIs versus tamoxifen on the risk of dementia. Therefore, using routinely collected primary care data from the United Kingdom (UK), we performed a cohort study that aimed to compare the risk of incident dementia among postmenopausal breast cancer survivors prescribed AI therapy with those prescribed tamoxifen.

## Methods

### Data source

We used data from the Clinical Practice Research Datalink General Practice Online Data (CPRD GOLD) primary care database of anonymised electronic health records (EHRs), which covers approximately 7% of the UK population, and to which 674 general practices across the UK have contributed patient data [17, 18]. The database enables long-term follow-up of population-based cohorts for pharmacoepidemiological research. Information is recorded by general practitioners and other primary care health professionals as part of routine patient care under the UK’s National Health Service, which provides universal free healthcare. The data recorded includes patient demographics, clinical symptoms, diagnoses, and referrals to secondary care, which are entered using Read codes (coded clinical terminology used for recording in the UK) [19] as well as all prescriptions issued. Information from secondary care sent by emails and letters is also recorded. Diagnoses in CPRD GOLD have generally been found to have high validity, [17] including for dementia diagnoses, which have an 83–84% positive predictive value (PPV) [20, 21]. In the UK, dementia is largely managed in primary care, making CPRD GOLD an appropriate setting for capturing dementia cases. Through linkage to UK cancer registrations, over 90% of breast cancer diagnoses in CPRD GOLD have been validated, and over 90% percent of breast cancer registrations are captured in CPRD GOLD [22]. The database is broadly representative of the UK demographic in terms of age, sex, and ethnicity [23]. The study protocol was approved by the Independent Scientific Advisory Committee for Medicines and Healthcare products Regulatory Agency (reference 17–122), and ethical approval for this study was obtained from The LSHTM Research Ethics Committee (MSc Ethics Ref: 13465).

### Study population

**Fig. 1:**
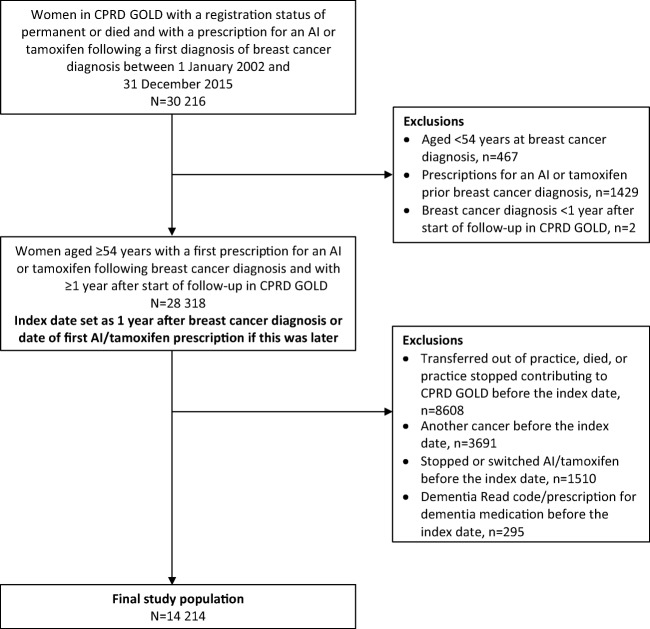
Identification of the postmenopausal breast cancer study population. AI, aromatase inhibitor; Clinical Practice Research Datalink (CPRD) GOLD

Identification of the study population is depicted in the Fig. 1. We included all women in CPRD GOLD with a permanent registration status, aged at least 54 years (median age of natural menopause in European women) [24], and with a first prescription for an AI (anastrozole, exemestane, or letrozole) or tamoxifen following a first recorded diagnosis of breast cancer between 1 January 2002 and 31 December 2015. Although AIs were not advocated by the National Institute for Health and Care Excellence until 2006 [25], preliminary analysis indicated widespread use of these drugs in preceding years. Although oestrogen-receptor positive status of breast cancer is not available in the database, women were assumed to have oestrogen-receptor positive disease because this is the indication for endocrine therapy. Women were also required to have at least 12-month follow-up in the database before their first breast cancer diagnosis and to be still alive and registered with the general practice 1-year post-diagnosis. The index date was 1 year following breast cancer diagnosis or the date of first prescription for endocrine therapy (AI/tamoxifen) if this came later; the rationale was to minimize the possibility that any immediate effects on these outcomes would have been due to initial chemotherapy/radiotherapy treatment as well as to minimize the possibility of including prevalent cases of dementia. Patients whose index date occurred after the end of the study period were excluded. Women were also excluded if they had any of the following before the index date: a Read code for dementia (Appendix Table 1); a dementia-specific medication prescription (Appendix Table 2); or a recorded diagnosis of a different cancer.

### Exposure to AI/tamoxifen therapy

In the UK, the recommended duration of use for AIs during the period of this study was 5 years or 3 years as part of a sequential regimen following 2-year tamoxifen treatment [25]. Endocrine therapy was categorized according to initial treatment prescribed, either AI or tamoxifen. If a woman switched from tamoxifen to an AI, they moved into a separate exposure category “AI preceded by tamoxifen” on the date of their first AI prescription, while women who switched from an AI to tamoxifen moved into an exposure category “tamoxifen preceded by AI” on the date of their first tamoxifen prescription. Women were still able to contribute person-time to exposure categories if they had a gap in treatment and later restarted on either the same medication or switched treatment. A stop in treatment was defined as no prescription within 30 days after the end of the previous prescription.

### Follow-up and dementia outcomes

The primary outcome of interest was any type of incident dementia, defined as a first record of a prescription for a dementia-specific medication (Appendix Table 2) or Read code indicative of incident dementia (Appendix Table 3). Where possible, based on specific Read codes (Appendix Table 4), dementia cases were classed according to subtype—Alzheimer’s disease, vascular dementia, dementia with Lewy bodies, or mixed dementia—otherwise they were classed as unknown type. Follow-up began at the index date and ended at the earliest of incident dementia as defined above, death, transfer out of the practice, or end of the study period (31 December 2015).

### Covariates

Variables evaluated as potential confounders included age at the start of endocrine therapy (< 75 years and ≥ 75 years), practice level index of multiple deprivation (IMD)—a measure of socioeconomic deprivation (five quartiles with 1 being the least deprived and 5 being the most deprived) [23], smoking (never, current, ex-smoker, or unknown), body mass index (BMI in kg/m^2^; < 18, 18 to 25, 25 to < 30, ≥ 30, or unknown), alcohol consumption (never, current [light, moderate, heavy, or unknown quantity], ex-drinker, and unknown), diabetes (type I or II), hypertension, cardiovascular disease (CVD), cerebrovascular disease, hyperlipidaemia, depression, use of hormone replacement therapy (HRT), and time since the index date (time-updated; < 1 year, 1 to < 2 years; 2 to < 5 years, 5 to < 10 years, and ≥ 10 years). For lifestyle variables, we used the most recently recorded status/value before the index date.

### Statistical analysis

Baseline variables were described according to initial endocrine therapy prescribed, using frequency counts and percentages for categorical variables and means with standard deviation (SD) for continuous variables. Mantel-Haenszel stratification was used to calculate the crude incidence rate ratio with 95% confidence interval (95% CI) for incident dementia comparing use of AIs with tamoxifen (reference group) as first endocrine therapy. Cox regression (using age as the timescale) was used with AI exposure as a time-updated variable to quantify the association between use of AIs (vs. tamoxifen) and incident dementia, adjusted for confounders. Hazard ratios (HRs) with 95% CIs were calculated for any type of dementia as the primary outcome, and for Alzheimer’s disease and vascular dementia as secondary outcomes (because of their different aetiologies, it is possible that an association between AIs and dementia could vary between subtypes). The main comparison of interest was between person-time on an AI as first endocrine therapy (prior to any switch) and person-time on tamoxifen as first endocrine therapy (prior to any switch). In the Cox regression modelling, each potential confounder was added to the attained-age adjusted model one at a time, i.e. a series of 2-variable models, with a change in the HR of 10% or more compared with the age-only model deemed to provide evidence of confounding by the added variable. For variables with missing data (smoking, alcohol consumption, and BMI), potential confounding for each was explored using a complete case approach (excluding patients with missing data on that particular variable). A sensitivity analyses was performed, restricting to dementia cases identified on the basis of a Read code (i.e. those with only a dementia medication prescription during follow-up were deemed non-cases). Potential effect modification by age and previous use of HRT was investigated. Stata 14 (Stata Corp, Texas) was used for all analyses.

## Results

### Study population and baseline characteristics

We identified 14,214 postmenopausal women with a first prescription for an AI or tamoxifen following a first breast cancer diagnosis. Among these, 8018 (56.4%) women started endocrine treatment with an AI and 6196 (43.6%) started with tamoxifen. Baseline characteristics of the study population are presented in Table 1. Compared with women starting on tamoxifen, women starting treatment with an AI were slightly older at start of follow-up and were more likely to start therapy in later calendar years. They were also more likely to reside in a deprived area, be obese, and have a previous record of hypertension, diabetes, CVD, cerebrovascular disease, and depression, but were less likely to be current smokers or alcohol drinkers, or to have previously received HRT. The total number of women ever prescribed an AI was 10,385 (73.1%). Among ever AI users, the majority were prescribed an AI as initial endocrine therapy (77.2%, *n* = 8018), with less than a quarter (22.8%, *n* = 2367) receiving an AI as part of a sequential regimen following initial tamoxifen therapy. Median follow-up was 2.5 years among women starting treatment on an AI and 4.8 years among women starting on tamoxifen; maximum follow-up times in the two groups were 12.9 years and 13.0 years respectively. Overall, 1222 patients (8.6%) had missing data on at least one lifestyle variable.

**Table 1:** Baseline characteristics of postmenopausal 1-year breast cancer survivors prescribed AI/tamoxifen, overall, and by initial treatment

Characteristic	Patients starting with an AI (*N* = 8018)	Patients starting with tamoxifen (*N* = 6196)	Overall study population (*N* = 14,214)
Age (years)			
55–64	2695 (33.6)	2568 (41.5)	5263 (37.0)
65–74	2629 (32.8)	2131 (34.4)	4760 (33.5)
≥ 75	2694 (33.6)	1497 (24.2)	4191 (29.5)
Mean (SD)	70.9 (9.9)	68.6 (9.4)	69.9 (9.7)
Median (IQR)	69.2 (62.9–78.6)	67.2 (61.2–74.6)	68.3 (62.1–77.0)
Calendar year			
2003–2006	942 (11.8)	2886 (46.6)	3828 (26.9)
2007–2009	2049 (25.6)	1606 (25.9)	3655 (25.7)
2010–2012	2634 (32.9)	967 (15.6)	3601 (25.3)
2013–2015	2393 (29.9)	737 (11.9)	3130 (22.0)
Practice-level IMD			
1 (least deprived)	1768 (22.1)	1216 (19.6)	2984 (21.0)
2	1620 (20.2)	1305 (21.1)	2925 (20.6)
3	1629 (20.3)	1422 (23.0)	3051 (21.5)
4	1619 (20.2)	1210 (19.5)	2829 (19.9)
5 (most deprived)	1382 (17.2)	1043 (16.8)	2425 (17.1)
BMI (kg/m^2^)^*^			
Underweight (< 18)	95 (1.3)	73 (1.2)	168 (1.3)
Healthy weight (18 to < 25)	2417 (31.8)	2190 (37.3)	4607 (34.2)
Overweight (25 to < 30)	2718 (35.8)	2068 (35.2)	4786(35.5)
Obese (≥ 30)	2366 (31.2)	1540 (26.2)	3906 (29.0)
Unknown	422 (5.3)	325 (5.2)	747 (5.3)
Mean (SD)	28.0 (5.8)	27.2 (5.4)	27.7 (5.6)
Median (IQR)	27.1 (24.0–31.2)	26.4 (23.4–30.1)	26.8 (23.7–30.8)
Smoking^*^			
Never	3099 (38.8)	2466 (40.0)	5565 (39.3)
Current	834 (10.4)	874 (14.2)	1708 (12.1)
Former	4065 (50.8)	2833 (45.9)	6898 (48.7)
Unknown	20 (0.3)	23 (0.4)	43 (0.3)
Alcohol consumption^*^			
Never	1016 (13.5)	833 (14.3)	1849 (13.9)
Current drinker			
Low	4485 (59.7)	3649 (62.7)	8134 (61.0)
Medium	331 (4.4)	251 (4.3)	582 (4.4)
Heavy	98 (1.3)	82 (1.4)	180 (1.4)
Unknown	531 (7.1)	368 (6.3)	899 (6.7)
Ex-drinker	1055 (14.0)	640 (11.0)	1695 (12.7)
Unknown	502 (6.3)	373 (6.0)	875 (6.2)
Comorbidity/comedication^*^			
Hypertension	4019 (50.1)	2621 (42.3)	6640 (46.7)
Diabetes	998 (12.5)	557 (9.0)	1555 (10.4)
Hyperlipidaemia	1662 (20.7)	958 (15.5)	2620 (18.4)
CVD	1837 (22.9)	866 (14.0)	2703 (19.0)
CeVD	382 (4.8)	129 (2.1)	511 (3.6)
Depression	2473 (30.8)	1700 (27.4)	4173 (29.4)
HRT	2767 (34.5)	2340 (37.8)	5107 (35.9)

^*^Ever before the index date (for lifestyle variables, the most recent value/status was used). *AI* aromatase inhibitor, *BMI* body mass index, *CeVD* cerebrovascular disease, *CVD* cardiovascular disease, *HRT* hormone replacement therapy, *IMD* index of multiple deprivation

A total of 368 incident cases of dementia (median age 82.7 years, IQR 77.0–87.2) were identified during 57,102 person-years of follow-up. Alzheimer’s disease accounted for over a third of cases (34.5%, *n* = 127; median age 79.4, IQR 73.5–85.5) and vascular dementia for a quarter (24.5%, *n* = 90; median age 82.7, IQR 77.0–87.2). There was one case of dementia with Lewy bodies (0.3%), seven cases of mixed dementia (1.9%), and 143 cases had unknown dementia subtype (38.9%; median age 82.7 years, IQR 77.0 to 87.2). The crude incidence rate of dementia was 7.46 per 1000 AI exposed person-years (95% CI 6.43–8.65) among women on an AI as their first endocrine treatment, and 6.32 per 1000 tamoxifen exposed person-years (95% CI 5.34–7.47) among women on tamoxifen as their first endocrine treatment. In unadjusted analyses, no association was seen between AI use (vs tamoxifen) and dementia incidence (crude incidence rate ratio comparing AI vs tamoxifen as first endocrine therapy, 1.18 (95% CI 0.94–1.45). Age-adjusted HRs for the association between AI exposure and other explanatory variables with incident dementia are shown in Table 2. After adjustment for age, the point estimate for the association between AI (vs. tamoxifen) and dementia risk moved closer still to the null; HR 1.04 (95% CI 0.83–1.30). The age-adjusted HR was not changed by ≥ 10% by any other potential confounder or following adjustment for all potential confounders (fully adjusted model, HR 1.08, 95% CI 0.84–1.38) (Table 3). Therefore, the age-adjusted model was retained as the final Cox model. In the sensitivity analysis restricting dementia cases to those with an incident dementia code during follow-up (excluding 18 cases with only a dementia medication prescription), the findings for the risk of dementia were not materially different from those in the main analysis; HR 1.06 (95% CI 0.82–1.37).

**Table 2:** Incidence rates and adjusted HRs (with 95% CIs) for the association between AI exposure (and other explanatory variables) and incident dementia among postmenopausal 1-year breast cancer survivors prescribed AI/tamoxifen

Explanatory variable	Incident dementia cases, *n* *N* = 368	Total person-years (1000s)	Crude incidence rate of dementia per 1000 person-years (95% CI)	Age-adjusted HR^a^	*p* value^*^
AI exposure					
Tamoxifen as first endocrine therapy	136	21.54	6.32 (5.34–7.47)	1.0 (reference)	
AI as first endocrine therapy	175	23.46	7.46 (6.43–8.65)	1.04 (0.83–1.30)	
AI with prior tamoxifen use	43	10.48	4.10 (3.04–5.53)	0.73 (0.52–1.04)	
Tamoxifen with prior AI use	14	1.62	8.63 (5.11–14.57)	1.11 (0.64–1.93)	0.186
Age at index date (years)					
55–64	27	24.27	1.11 (0.76–1.62)	1.0 (reference)	
65–74	93	19.74	4.71 (3.84–5.77)	0.88 (0.42–1.67)	
≥ 75	248	13.08	18.96 (16.74–21.47)	0.87 (0.41–1.84)	0.9264
Calendar year					
2003–2006	174	23.93	7.27 (6.27–8.44)	1.0 (reference)	
2007–2009	88	17.85	4.93 (4.00–6.08)	0.75 (0.58–0.96)	
2010–2012	87	11.34	7.67 (6.22–9.46)	1.19 (0.92–1.54)	
2013–2015	19	3.98	4.77 (3.04–7.48)	0.79 (0.49–1.27)	0.0136
IMD (practice level)					
1 (least deprived)	70	12.29	5.70 (4.51–7.20)	1.0 (reference)	
2	79	11.72	6.74(5.41–8.40)	1.16 (0.84–1.60)	
3	72	12.23	5.89 (4.67–7.42)	0.90 (0.65–1.25)	
4	69	11.16	6.18 (4.88–7.83)	1.02 (0.73–1.42)	
5 (most deprived)	78	9.70	8.04 (6.44–10.04)	1.32 (0.96–1.83)	0.158
BMI (kg/m^2^)					
Underweight (< 18)	10	0.51	19.80 (10.65–36.80)	1.53 (0.80–2.92)	
Healthy weight (18 to < 25)	142	19.01	7.47 (6.34–8.81)	1.0 (reference)	
Overweight (25 to < 30)	118	19.86	5.94 (4.96–7.12)	0.76 (0.60–0.98)	
Obese (≥ 30)	74	15.49	4.78 (3.80–6.00)	0.76 (0.57–1.01)	0.035
Smoking					
Never	148	22.87	6.47 (5.51–7.60)	1.0 (reference)	
Current	40	7.73	5.18 (3.80–7.06)	1.14 (0.80–1.62)	
Former	178	26.45	6.73 (5.81–7.79)	1.14 (0.92–1.42)	0.467
Alcohol consumption					
Never	71	7.75	9.16 (7.26–11.56)	1.0 (reference)	
Current low	178	33.96	5.24 (4.53–6.07)	0.81 (0.62–1.07)	
Current medium	10	2.37	4.21 (2.27–7.83)	0.92 (0.47–1.79)	
Current high	4	0.61	6.58 (2.47–17.54)	1.29 (0.47–3.53)	
Unknown	28	3.46	8.10(5.59–11.73)	1.13 (0.73–1.75)	
Ex-drinker	54	6.03	8.96 (6.86–11.69)	1.01 (0.71–1.44)	0.391
Diabetes					
No	320	51.77	6.18 (5.54–6.90)	1.0 (reference)	
Yes	48	5.33	9.01 (6.79–11.95)	1.09 (0.81–1.48)	0.5661
Hypertension					
No	156	31.77	4.91 (4.20–5.75)	1.0 (reference)	
Yes	212	25.34	8.37 (7.31–9.57)	0.96 (0.78–1.19)	0.705
CeVD					
No	339	55.66	6.09 (5.48–6.77)	1.0 (reference)	
Yes	29	1.44	20.16 (14.01–29.01)	1.57 (1.07–2.30)	0.0301
CVD					
No	254	47.50	5.35 (4.73–6.05)	1.0 (reference)	
Yes	114	9.60	11.87 (9.88–14.27)	1.22 (0.98–1.53)	0.0839
Hyperlipidaemia					
No	288	47.42	6.07 (5.41–6.82)	1.0 (reference)	
Yes	80	9.69	8.26 (6.63–10.28)	1.16 (0.90–1.49)	0.2546
Depression					
No	262	41.33	6.34 (5.62–7.15)	1.0 (reference)	
Yes	106	15.77	6.72 (5.56–8.13)	1.26 (1.01–1.58)	0.0472
HRT					
No	294	35.38	8.31 (7.41–9.32)	1.0 (reference)	
Yes	74	21.72	3.41 (2.71–4.28)	0.98 (0.75–1.29)	0.900

^a^Significance testing was performed using the likelihood ratio test

*AI* aromatase inhibitor, *BMI* body mass index, *CI* confidence interval, *CVD* cardiovascular disease, *CeVD* cerebrovascular disease, *HR* hazard ratio, *HRT* hormone replacement therapy, *IMD* index of multiple deprivation, *RR* rate ratio

**Table 3:** HRs (95% CI) for the association between AI (vs. tamoxifen) and incident dementia among postmenopausal 1-year breast cancer survivors prescribed AI/tamoxifen, adjusted for potential confounders

	HR for dementia (95% CI)^*^
Adjusted for attained age only (final model)	1.04 (0.83–1.03)
Adjusted for attained age plus	
Age at index date	1.05 (0.84–1.32)
Calendar year	1.10 (0.86–1.41)
IMD (practice-level)	1.04 (0.83–1.31)
BMI^†^	1.07 (0.85–1.36)
Smoking^†^	1.04 (0.83–1.30)
Alcohol consumption^†^	1.05 (0.83–1.33)
CVD^‡^	1.02 (0.82–1.28)
CeVD^‡^	1.02 (0.81–1.28)
Hypertension^‡^	1.05 (0.84–1.31)
Hyperlipidaemia^‡^	1.03 (0.82–1.29)
Diabetes^‡^	1.04 (0.83–1.30)
Depression^‡^	1.03 (0.83–1.29)
HRT use^‡^	1.04 (0.83–1.30)
Time since index date	1.08 (0.86–1.93)
All the above variables	1.08 (0.84–1.38)

^*^From Cox regression with age as timescale; AI vs. tamoxifen exposure. ^†^Using the nearest value/status before the index date. ^‡^Recorded any time before the index date

*AI* aromatase inhibitor, *BMI* body mass index, *CeVD* cerebrovascular disease, *CVD* cardiovascular disease, *CI* confidence interval, *HR* hazard ratio, *HRT* hormone replacement therapy, *IMD* Index of Multiple Deprivation

There was no evidence that the risk of dementia with AI therapy (vs. tamoxifen) was modified by age at start of therapy (*p* = 0.265) or previous HRT use (*p* = 0.661) (Table 4). Testing of non-proportional hazards indicated there was no interaction with attained age (< 74 years or ≥ 75 years). In secondary analyses, there was no evidence to suggest that AI exposure (vs. tamoxifen) was associated with a different rate of either Alzheimer’s disease or vascular dementia; age-adjusted HRs were 0.89 (95% CI 0.61–1.31) for Alzheimer’s and 0.94 (95% CI 0.59–1.50) for vascular dementia.

**Table 4:** HRs (95% CI) for the association between AI exposure and incidence dementia among postmenopausal 1-year breast cancer survivors prescribed AI/tamoxifen, by age at first endocrine therapy prescription, and by previous HRT use

Age at first AI/tamoxifen prescription (years)	Incident dementia cases, *N*	Total person-years (1000s)	Incidence rate per 1000 person-years (95% CI)	HR (95% CI)^*^
55–74				
Tamoxifen as first endocrine therapy	61	17.29	3.53 (2.74–4.53)	1.0 (reference)
AI as first endocrine therapy	47	17.31	2.72 (2.04–3.61)	0.85 (0.58–1.25)
AI with prior tamoxifen use	22	9.19	2.39 (1.58–3.64)	0.59 (0.36–0.97)
Tamoxifen with prior AI use	2	1.20	1.67 (0.42–6.68)	0.48 (0.12–1.98)
≥ 75				
Tamoxifen as first endocrine therapy	75	4.24	17.67 (14.09–22.16)	1.0 (reference)
AI as first endocrine therapy	128	6.15	20.80 (17.49–24.74)	1.19 (0.89–1.58)
AI with prior tamoxifen use	21	1.30	16.21 (10.57–24.86)	0.87 (0.53–1.41)
Tamoxifen with prior AI use	12	0.43	28.23 (16.03–49.71)	1.48 (0.80–2.72)
No previous HRT use				
Tamoxifen as first endocrine therapy	109	13.54	8.05 (6.67–9.71)	1.0 (reference)
AI as first endocrine therapy	143	15.04	9.51 (8.07–11.20)	1.04 (0.81–1.34)
AI with prior tamoxifen use	33	5.88	5.61 (4.00–7.89)	0.78 (0.53–1.15)
Tamoxifen with prior AI use	9	0.92	9.80 (5.10–18.83)	0.93 (0.47–1.83)
Previous HRT use				
Tamoxifen as first endocrine therapy	27	8.00	3.38 (2.32–4.92)	1.0 (reference)
AI as first endocrine therapy	32	8.42	3.80 (2.69–5.37)	1.04 (0.62–1.73)
AI with prior tamoxifen use	10	4.60	2.17 (1.17–4.04)	0.62 (0.30–1.28)
Tamoxifen with prior AI use	5	0.70	7.10 (2.96–17.06)	1.74 (0.67–4.52)

*p* value for interaction with age at first AI/tamoxifen prescription was 0.265; *p* value for interaction with HRT prescription was 0.661

*AI* aromatase inhibitor, *HR* hazard ratio, *HRT* hormone replacement therapy

^*^From Cox regression with age as timescale

## Discussion

In this large population-based cohort study with up to 13 years’ follow-up, we found no evidence that postmenopausal breast cancer survivors receiving treatment with an AI had a higher risk of incident dementia compared with those on tamoxifen. This finding was also seen for both Alzheimer’s disease and vascular dementia.

We are unaware of other studies that have directly compared the effect of AIs and tamoxifen on the risk of dementia among postmenopausal breast cancer patients in any setting. Our findings are, however, in line with those from a meta-analysis of six small cross-sectional studies by Underwood et al., who found no differences between AIs and tamoxifen among cognitive performance in breast cancer patients [11]. It should be noted that our findings in relation to dementia risk do not exclude the possibility that AIs and tamoxifen have differential effects on specific cognitive domains as tested among patients in cognition sub-studies of RCTs. The Breast International Group-98 (BIG-98) RCT sub-study [16] found better mean composite cognitive scores at trial completion among letrozole than tamoxifen arms, yet the absence of baseline cognitive assessment means the analysis was essentially cross-sectional. In the Tamoxifen and Exemestane Adjuvant Multinational (TEAM) RCT sub-study [8], AI users had significantly better executive functioning than tamoxifen users overall and significantly better information processing speed among women aged > 65 years but no differences in other cognitive domains.

Strengths of our study include the large sample from a validated data source representative of the UK demographic, and which has a high PPV for recorded dementia diagnoses [20, 21] and high validity and completeness of recorded breast cancer diagnoses [22]. We observed little confounding, yet residual confounding cannot be excluded. In particular, chemotherapy has been linked to cognitive impairment in breast cancer patients [26, 27] but could not be evaluated because this information is not captured well in CPRD GOLD. Inability to adjust for any potential imbalances in chemotherapy between AI and tamoxifen-only exposed patients could have biased the results, potentially in either direction. As information on stage and grade of breast cancer is also not systematically recorded in the database, we were also unable to adjust for any potential confounding effect this may have had if this was related to both dementia incidence and the choice of endocrine therapy. Lack of information on oestrogen-receptor positive status of the breast cancer meant that we were unable to identify women eligible for, but who did not receive, endocrine therapy and compare their risk of dementia with that among AI/tamoxifen exposed groups. These comparisons may have helped assess the individual causal effects of each drug. We also decided not to include comparative data from healthy controls because of the likelihood of confounding by factors related to breast cancer risk. We were only able to investigate diagnosed dementia; therefore, undiagnosed cases will have been missed. Any misclassification of dementia cases is likely to have been non-differential between exposure groups, which may have biased results towards the null. As CPRD GOLD captures all prescriptions issued in general practice, misclassification of AI and tamoxifen exposure is likely minimal, although prescriptions issued in secondary care may have been missed. Whether prescriptions are filled and medications are taken is unknown, yet both would be expected among women with a condition as serious as breast cancer.

Further evaluation of the cognitive safety of adjuvant endocrine breast cancer therapy in other large population-based cohorts is needed to corroborate our findings, including exploration of possible age-related and treatment duration effects, as well as potential effects of previous tamoxifen treatment. It is also important to note that our findings do not rule out the possibility that both AIs and tamoxifen are detrimental in terms of dementia risk compared with no endocrine therapy. Tamoxifen has oestrogen antagonist properties in some tissue (e.g. breast) and agonist properties in others (e.g. bone) but its action in the brain is unknown and it could potentially have an independent causal association with dementia risk. Among cognition RCT sub-studies and the few large observational studies on this topic, findings have been mixed, and comparisons are limited by heterogeneity in the study population, the exposure and comparison groups, and the specific cognitive outcome(s) evaluated [8–10, 12].

Dementia is a common disease, with an estimated prevalence of 7% among the general population aged over 65 years [28]; therefore, any effect of drug treatments on risk would be of public health importance. There is a need for further work involving separate evaluation of AIs and tamoxifen compared with no endocrine treatment to rule out our study’s null finding being a result of similar drug effects in both drug classes. Further research into the effect AIs have on anatomy, physiology, and metabolism, and on clinical events such as stroke, would also help better understand any effects on biological mechanisms linked to dementia risk and help build a more comprehensive understanding in this complex field.

## Electronic supplementary material

ESM 1 (DOCX 78 kb)

ESM 2 (DOCX 86 kb)

ESM 3 (DOCX 77 kb)

ESM 4 (DOCX 80 kb)
