# Correction to: Risk of dementia among postmenopausal breast cancer survivors treated with aromatase inhibitors versus tamoxifen: a cohort study using primary care data from the UK

**DOI:** 10.1007/s11764-019-00806-5

**Published:** 2019-10-22

**Authors:** Susan E. Bromley, Anthony Matthews, Liam Smeeth, Susannah Stanway, Krishnan Bhaskaran

**Affiliations:** 1 Department of Non-communicable Disease Epidemiology, London School of Hygiene and Tropical Medicine, London, UK; 2 EpiMed Communications Ltd, Abingdon, UK; 3 Royal Marsden NHS Foundation Trust, Sutton, Surrey, UK

**Correction to: Journal of Cancer Survivorship (2019) 13:632–640**

10.1007/s11764-019-00782-w

The article Risk of dementia among postmenopausal breast cancer survivors treated with aromatase inhibitors versus tamoxifen: a cohort study using primary care data from the UK, written by Susan E. Bromley, Anthony Matthews, Liam Smeeth, Susannah Stanway and Krishnan Bhaskaran was originally published electronically on the publisher’s internet portal (currently SpringerLink) on 18 July 2019 without open access. With the author(s)’ decision to opt for Open Choice the copyright of the article changed on August 2019 to © The Author(s) 2019 and the article is forthwith distributed under the terms of the Creative Commons Attribution 4.0 International License (http://creativecommons.org/licenses/by/4.0/), which permits use, duplication, adaptation, distribution and reproduction in any medium or format, as long as you give appropriate credit to the original author(s) and the source, provide a link to the Creative Commons license and indicate if changes were made.

