# Supplementary material for: Risk of dementia among postmenopausal breast cancer survivors treated with aromatase inhibitors versus tamoxifen: a cohort study using primary care data from the UK
Source: J Cancer Surviv. 2019 Jul 18;13(4):632–40. doi: 10.1007/s11764-019-00782-w (PMC6776493; doi:10.1007/s11764-019-00782-w)
Supplement: Supplementary file 1 — (DOCX 78 kb) [file 11764_2019_782_MOESM1_ESM.docx]

**Supplementary Table 1.** Read codes for prevalent/incident dementia.

| **Read code** | **Description** |
| --- | --- |
| e00..11 | senile dementia |
| e00..12 | senile/presenile dementia |
| e000.00 | uncomplicated senile dementia |
| e001.00 | presenile dementia |
| e001000 | uncomplicated presenile dementia |
| e001100 | presenile dementia with delirium |
| e001200 | presenile dementia with paranoia |
| e001300 | presenile dementia with depression |
| e001z00 | presenile dementia nos |
| e002.00 | senile dementia with depressive or paranoid features |
| e002000 | senile dementia with paranoia |
| e002100 | senile dementia with depression |
| e002z00 | senile dementia with depressive or paranoid features nos |
| e003.00 | senile dementia with delirium |
| e004.00 | arteriosclerotic dementia |
| e004.11 | multi infarct dementia |
| e004000 | uncomplicated arteriosclerotic dementia |
| e004100 | arteriosclerotic dementia with delirium |
| e004200 | arteriosclerotic dementia with paranoia |
| e004300 | arteriosclerotic dementia with depression |
| e004z00 | arteriosclerotic dementia nos |
| e012.00 | other alcoholic dementia |
| e012.11 | alcoholic dementia nos |
| e02y100 | drug-induced dementia |
| e041.00 | dementia in conditions ec |
| eu00.00 | [x]dementia in alzheimer's disease |
| eu00000 | [x]dementia in alzheimer's disease with early onset |
| eu00011 | [x]presenile dementia,alzheimer's type |
| eu00012 | [x]primary degen dementia, alzheimer's type, presenile onset |
| eu00013 | [x]alzheimer's disease type 2 |
| eu00100 | [x]dementia in alzheimer's disease with late onset |
| eu00111 | [x]alzheimer's disease type 1 |
| eu00112 | [x]senile dementia,alzheimer's type |
| eu00113 | [x]primary degen dementia of alzheimer's type, senile onset |
| eu00200 | [x]dementia in alzheimer's dis, atypical or mixed type |
| eu00z00 | [x]dementia in alzheimer's disease, unspecified |
| eu00z11 | [x]alzheimer's dementia unspec |
| eu01.00 | [x]vascular dementia |
| eu01.11 | [x]arteriosclerotic dementia |
| eu01000 | [x]vascular dementia of acute onset |
| eu01100 | [x]multi-infarct dementia |
| eu01111 | [x]predominantly cortical dementia |
| eu01200 | [x]subcortical vascular dementia |
| eu01300 | [x]mixed cortical and subcortical vascular dementia |
| eu01y00 | [x]other vascular dementia |
| eu01z00 | [x]vascular dementia, unspecified |
| eu02.00 | [x]dementia in other diseases classified elsewhere |
| eu02000 | [x]dementia in pick's disease |
| eu02100 | [x]dementia in creutzfeldt-jakob disease |
| eu02200 | [x]dementia in huntington's disease |
| eu02300 | [x]dementia in parkinson's disease |
| eu02400 | [x]dementia in human immunodef virus [hiv] disease |
| eu02500 | [x]lewy body dementia |
| eu02y00 | [x]dementia in other specified diseases classif elsewhere |
| eu02z00 | [x] unspecified dementia |
| eu02z11 | [x] presenile dementia nos |
| eu02z13 | [x] primary degenerative dementia nos |
| eu02z14 | [x] senile dementia nos |
| eu02z16 | [x] senile dementia, depressed or paranoid type |
| eu04100 | [x]delirium superimposed on dementia |
| eu10711 | [x]alcoholic dementia nos |
| f110.00 | alzheimer's disease |
| f110000 | alzheimer's disease with early onset |
| f110100 | alzheimer's disease with late onset |
| f111.00 | pick's disease |
| f116.00 | lewy body disease |
| fyu3000 | [x]other alzheimer's disease |
| 1461 | h/o: dementia |
| 3A...12 | Dementia assessment |
| 3AE..00 | Global deterioration scale: assessment of prim deg dementia |
| 66h..00 | Dementia monitoring |
| 6AB..00 | Dementia annual review |
| 8Hla.00 | Referral to dementia care advisor |
| 9hD..00 | Exception reporting: dementia quality indicators |
| 9hD0.00 | Excepted from dementia quality indicators: Patient unsuitabl |
| 9hD1.00 | Excepted from dementia quality indicators: Informed dissent |
| 9Ou..00 | Dementia monitoring administration |
| 9Ou1.00 | Dementia monitoring first letter |
| 9Ou2.00 | Dementia monitoring second letter |
| 9Ou3.00 | Dementia monitoring third letter |
| 9Ou4.00 | Dementia monitoring verbal invite |
| 9Ou5.00 | Dementia monitoring telephone invite |
| F11x.00 | Cerebral degeneration in other disease EC* |
| F11x200 | Cerebral degeneration due to cerebrovascular disease* |
| F11x900 | Cerebral degeneration in Parkinson's disease* |
| ZR1K.00 | Alzheimer's disease assessment scale |
| ZR1K.11 | ADAS - Alzheimer's disease assessment scale |
| ZR1T.00 | Arizona battery for communication disorders of dementia |
| ZR2X.12 | BDRS - Blessed dementia rating scale |
| ZR3V.00 | Clinical dementia rating scale |
| ZR3V.11 | DRS - Clinical dementia rating scale |
| ZR3V.12 | CDR - Clinical dementia rating scale |
| ZR3V.13 | Dementia rating scale |
