# Supplementary material for: Risk of dementia among postmenopausal breast cancer survivors treated with aromatase inhibitors versus tamoxifen: a cohort study using primary care data from the UK
Source: J Cancer Surviv. 2019 Jul 18;13(4):632–40. doi: 10.1007/s11764-019-00782-w (PMC6776493; doi:10.1007/s11764-019-00782-w)
Supplement: Supplementary file 2 — (DOCX 86 kb) [file 11764_2019_782_MOESM2_ESM.docx]

Supplementary Table 2. Codes for dementia-specific medications.

| **Product code** | **Multilex code** | **Strength** | **Form** | | **Drug substance** | | **Product name** | |
| --- | --- | --- | --- | --- | --- | --- | --- | --- |
| 33009 | !8503762 |  |  | |  | | rivastigmine | |
| 33007 | !8503760 |  |  | |  | | rivastigmine | |
| 33008 | !8503761 |  |  | |  | | rivastigmine | |
| 18800 | 84771020 | 10mg | Tablet | | memantine hydrochloride | | ebixa 10mg tablets (lundbeck ltd) | |
| 36976 | 94170020 | 4.6mg/24 Hours | | Transdermal Patch | | rivastigmine | rivastigmine 4.6mg/24hours transdermal patches | |
| 58780 | 15913021 | 9.5mg/24hour | Transdermal patch | | rivastigmine | | | voleze 9.5mg/24hours transdermal patches (focus pharmaceuticals ltd) |
| 10187 | 85756020 | 4mg | Tablet | | galantamine hydrobromide | | | galantamine 4mg tablets |
| 29288 | 86236020 | 4mg/1ml | Oral solution | | galantamine hydrobromide | | | reminyl 4mg/ml oral solution (shire pharmaceuticals ltd) |
| 55928 | 05472020 | 4.5mg | Capsule | | rivastigmine hydrogen tartrate | | | exelon 4.5mg capsules (waymade healthcare plc) |
| 37188 | 92625020 | 10mg | Orodispersible Tablet | | donepezil hydrochloride | | | aricept evess 10mg orodispersible tablets (eisai ltd) |
| 56771 | 41393020 | 3mg | Capsule | | rivastigmine hydrogen tartrate | | | rivastigmine 3mg capsules (dr reddy's laboratories (uk) ltd) |
| 57171 | 16510021 | 9.5mg/24hour | Transdermal patch | | rivastigmine | | | erastig 9.5mg/24hours transdermal patches (teva uk ltd) |
| 58937 | 16263021 |  |  | |  | | | exelon 13.3mg/24hours transdermal patches (novartis pharmaceuticals uk ltd) |
| 56709 | 45236020 | 16mg | Modified-release capsule | | galantamine hydrobromide | | | gatalin xl 16mg capsules (aspire pharma ltd) |
| 56600 | 42200020 | 5mg | Tablet | | donepezil hydrochloride | | | donepezil 5mg tablets (zentiva) |
| 61476 | 40751020 | 24mg | Modified-release capsule | | galantamine hydrobromide | | | acumor xl 24mg capsules (mylan ltd) |
| 61385 | 16813021 | 10mg | Tablet | | memantine hydrochloride | | | nemdatine 10mg tablets (actavis uk ltd) |
| 57627 | 16508021 | 4.6mg/24hour | Transdermal patch | | rivastigmine | | | erastig 4.6mg/24hours transdermal patches (teva uk ltd) |
| 58709 | 39844020 | 10mg | Tablet | | donepezil hydrochloride | | | donepezil 10mg tablets (a a h pharmaceuticals ltd) |
| 60493 | 21925021 | 24mg | Modified-release capsule | | galantamine hydrobromide | | | galantex xl 24mg capsules (creo pharma ltd) |
| 59871 | 36322020 | 2mg/1ml | Oral suspension | | donepezil hydrochloride | | | donepezil 10mg/5ml oral suspension |
| 9854 | 76135020 | 4mg | Tablet | | galantamine hydrobromide | | | reminyl 4mg tablets (shire pharmaceuticals ltd) |
| 39240 | 95625020 | 20mg | Tablet | | memantine hydrochloride | | | memantine 20mg tablets |
| 11827 | 77544020 | 2mg/1ml | Oral solution | | rivastigmine hydrogen tartrate | | | rivastigmine 2mg/ml oral solution sugar free |
| 2931 | 84649020 | 10mg | Tablet | | donepezil hydrochloride | | | donepezil 10mg tablets |
| 60107 | 39838020 | 5mg | Tablet | | donepezil hydrochloride | | | donepezil 5mg tablets (alliance healthcare (distribution) ltd) |
| 53842 | 05435020 | 5mg | Tablet | | donepezil hydrochloride | | | aricept 5mg tablets (waymade healthcare plc) |
| 11546 | 80919020 | 1.5mg | Capsule | | rivastigmine hydrogen tartrate | | | exelon 1.5mg capsules (novartis pharmaceuticals uk ltd) |
| 61676 | 27172021 |  |  | |  | | | donepezil 1mg/ml oral solution sugar free |
| 48015 | 00321021 | 24mg | Modified-release capsule | | galantamine hydrobromide | | | galsya xl 24mg capsules (consilient health ltd) |
| 4597 | 58075020 | 1.5mg | Capsule | | rivastigmine hydrogen tartrate | | | rivastigmine 1.5mg capsules |
| 37957 | 94176020 | 9.5mg/24hour | Transdermal patch | | rivastigmine | | | exelon 9.5mg/24hours transdermal patches (novartis pharmaceuticals uk ltd) |
| 56631 | 16262021 |  |  | |  | | | rivastigmine 13.3mg/24hours transdermal patches |
| 11654 | 85757020 | 8mg | Tablet | | galantamine hydrobromide | | | galantamine 8mg tablets |
| 6225 | 80100020 | 10mg | Tablet | | memantine hydrochloride | | | memantine 10mg tablets |
| 62868 | 29831021 | 24mg | Modified-release capsule | | galantamine hydrobromide | | | gazylan xl 24mg capsules (teva uk ltd) |
| 63217 | 39839020 | 5mg | Tablet | | donepezil hydrochloride | | | donepezil 5mg tablets (a a h pharmaceuticals ltd) |
| 62164 | 24017021 | 9.5mg/24hour | Transdermal patch | | rivastigmine | | | alzest 9.5mg/24hours transdermal patches (dr reddy's laboratories (uk) ltd) |
| 62925 | 40750020 |  |  | |  | | | acumor xl 16mg capsules (mylan ltd) |
| 5334 | 76137020 | 12mg | Tablet | | galantamine hydrobromide | | | reminyl 12mg tablets (shire pharmaceuticals ltd) |
| 24088 | 89210020 | 24mg | Modified-release capsule | | galantamine hydrobromide | | | reminyl xl 24mg capsules (shire pharmaceuticals ltd) |
| 18556 | 77547020 | 2mg/1ml | Oral solution | | rivastigmine hydrogen tartrate | | | exelon 2mg/ml oral solution (novartis pharmaceuticals uk ltd) |
| 11752 | 58077020 | 4.5mg | Capsule | | rivastigmine hydrogen tartrate | | | rivastigmine 4.5mg capsules |
| 63405 | 00319021 | 16mg | Modified-release capsule | | galantamine hydrobromide | | | galsya xl 16mg capsules (consilient health ltd) |
| 7329 | 76502020 | 4mg/1ml | Oral solution | | galantamine hydrobromide | | | galantamine 20mg/5ml oral solution sugar free |
| 20404 | 80921020 | 4.5mg | Capsule | | rivastigmine hydrogen tartrate | | | exelon 4.5mg capsules (novartis pharmaceuticals uk ltd) |
| 11716 | 80920020 | 3mg | Capsule | | rivastigmine hydrogen tartrate | | | exelon 3mg capsules (novartis pharmaceuticals uk ltd) |
| 38976 | 95635020 | 5mg+10mg+15mg+20mg | Tablet | | memantine hydrochloride | | | memantine 5mg+10mg+15mg+20mg tablet |
| 61921 | 29832021 | 24mg | Modified-release capsule | | galantamine hydrobromide | | | luventa xl 24mg capsules (fontus health ltd) |
| 64982 | 15384021 | 20mg | Tablet | | memantine hydrochloride | | | memantine 20mg tablets (teva uk ltd) |
| 39362 | 95639020 | 5mg+10mg+15mg+20mg | Tablets | | memantine hydrochloride | | | ebixa tablets treatment initiation pack (lundbeck ltd) |
| 18587 | 89206020 | 8mg | Modified-release capsule | | galantamine hydrobromide | | | reminyl xl 8mg capsules (shire pharmaceuticals ltd) |
| 48443 | 41262020 |  |  | |  | | | donepezil 10mg orodispersible tablets |
| 35179 | 92619020 | 5mg | Orodispersible tablet | | donepezil hydrochloride | | | donepezil 5mg orodispersible tablets sugar free |
| 63360 | 29830021 | 16mg | Modified-release capsule | | galantamine hydrobromide | | | luventa xl 16mg capsules (fontus health ltd) |
| 55720 | 45237020 | 24mg | Modified-release capsule | | galantamine hydrobromide | | | gatalin xl 24mg capsules (aspire pharma ltd) |
| 35088 | 92621020 | 10mg | Orodispersible Tablet | | donepezil hydrochloride | | | donepezil 10mg orodispersible tablets sugar free |
| 53922 | 41263020 |  |  | |  | | | donepezil 10mg orodispersible tablets (consilient health ltd) |
| 62780 | 24016021 | 4.6mg/24hour | Transdermal patch | | rivastigmine | | | alzest 4.6mg/24hours transdermal patches (dr reddy's laboratories (uk) ltd) |
| 37444 | 94174020 | 4.6mg/24 Hours | Transdermal Patch | | rivastigmine | | | exelon 4.6mg/24hours transdermal patches (novartis pharmaceuticals uk ltd) |
| 20140 | 89208020 | 16mg | Modified-release capsule | | galantamine hydrobromide | | | reminyl xl 16mg capsules (shire pharmaceuticals ltd) |
| 56421 | 45238020 |  |  | |  | | | gatalin xl 8mg capsules (aspire pharma ltd) |
| 5247 | 84654020 | 10mg | Tablet | | donepezil hydrochloride | | | aricept 10mg tablets (eisai ltd) |
| 10255 | 89200020 | 8mg | Modified Release Capsules | | galantamine hydrobromide | | | galantamine 8mg modified-release capsules |
| 48442 | 41264020 |  |  | |  | | | donepezil 5mg orodispersible tablets |
| 63226 | 29518021 | 9.5mg/24hour | Transdermal patch | | rivastigmine | | | prometax 9.5mg/24hours transdermal patches (novartis pharmaceuticals uk ltd) |
| 5616 | 80912020 | 6mg | Capsule | | rivastigmine hydrogen tartrate | | | exelon 6mg capsules (novartis pharmaceuticals uk ltd) |
| 48482 | 00317021 | 8mg | Modified-release capsule | | galantamine hydrobromide | | | galsya xl 8mg capsules (consilient health ltd) |
| 9786 | 76950020 | 6mg | Capsule | | rivastigmine hydrogen tartrate | | | rivastigmine 6mg capsules |
| 11751 | 58076020 | 3mg | Capsule | | rivastigmine hydrogen tartrate | | | rivastigmine 3mg capsules |
| 57139 | 10541020 | 10mg | Tablet | | memantine hydrochloride | | | ebixa 10mg tablets (de pharmaceuticals) |
| 58969 | 16509021 |  |  | |  | | | rivastigmine 4.6mg/24hours transdermal patches (a a h pharmaceuticals ltd) |
| 11635 | 85758020 | 12mg | Tablet | | galantamine hydrobromide | | | galantamine 12mg tablets |
| 18062 | 76136020 | 8mg | Tablet | | galantamine hydrobromide | | | reminyl 8mg tablets (shire pharmaceuticals ltd) |
| 63951 | 21545021 | 9.5mg/24hour | Transdermal patch | | rivastigmine | | | rivastigmine 9.5mg/24hours transdermal patches (actavis uk ltd) |
| 5400 | 84653020 | 5mg | Tablet | | donepezil hydrochloride | | | aricept 5mg tablets (eisai ltd) |
| 11837 | 80102020 | 10mg/1ml | Oral solution | | memantine hydrochloride | | | memantine 10mg/ml oral solution sugar free |
| 39363 | 95627020 | 20mg | Tablet | | memantine hydrochloride | | | ebixa 20mg tablets (lundbeck ltd) |
| 37132 | 94172020 | 9.5mg/24hour | Transdermal patch | | rivastigmine | | | rivastigmine 9.5mg/24hours transdermal patches |
| 7361 | 89204020 | 24mg | Modified-release capsule | | galantamine hydrobromide | | | galantamine 24mg modified-release capsules |
| 58947 | 39842020 | 10mg | Tablet | | donepezil hydrochloride | | | donepezil 10mg tablets (accord healthcare ltd) |
| 53882 | 43884020 | 2mg/1ml | Oral solution | | rivastigmine hydrogen tartrate | | | rivastigmine 2mg/ml oral solution |
| 14309 | 89202020 | 16mg | Modified Release Capsules | | galantamine hydrobromide | | | galantamine 16mg modified-release capsules |
| 36848 | 92623020 | 5mg | Orodispersible tablet | | donepezil hydrochloride | | | aricept evess 5mg orodispersible tablets (eisai ltd) |
| 9966 | 55435020 | 10mg/1ml | Oral solution | | memantine hydrochloride | | | ebixa 5mg/0.5ml pump actuation oral solution (lundbeck ltd) |
| 62867 | 29829021 | 16mg | Modified-release capsule | | galantamine hydrobromide | | | gazylan xl 16mg capsules (teva uk ltd) |
| 60723 | 47400020 | 6mg | Capsule | | rivastigmine hydrogen tartrate | | | rivastigmine 6mg capsules (waymade healthcare plc) |
| 61618 | 17039021 | 20mg | Tablet | | memantine hydrochloride | | | nemdatine 20mg tablets (actavis uk ltd) |
| 2930 | 84648020 | 5mg | Tablet | | donepezil hydrochloride | | | donepezil 5mg tablets |
| 65333 | 20305021 | 10mg/1ml | Oral solution | | memantine hydrochloride | | | memantine 10mg/ml oral solution sugar free (alliance healthcare (distribution) ltd) |
