# Supplementary material for: Risk of dementia among postmenopausal breast cancer survivors treated with aromatase inhibitors versus tamoxifen: a cohort study using primary care data from the UK
Source: J Cancer Surviv. 2019 Jul 18;13(4):632–40. doi: 10.1007/s11764-019-00782-w (PMC6776493; doi:10.1007/s11764-019-00782-w)
Supplement: Supplementary file 4 — (DOCX 80 kb) [file 11764_2019_782_MOESM4_ESM.docx]

**Supplementary Table 4**. Read codes for dementia subtypes.

| **Read code** | **Description** | **Dementia subtype** |
| --- | --- | --- |
| eu00.00 | [x]dementia in alzheimer's disease | Alzheimer’s disease |
| eu00000 | [x]dementia in alzheimer's disease with early onset | Alzheimer’s disease |
| eu00011 | [x]presenile dementia,alzheimer's type | Alzheimer’s disease |
| eu00012 | [x]primary degen dementia, alzheimer's type, presenile onset | Alzheimer’s disease |
| eu00013 | [x]alzheimer's disease type 2 | Alzheimer’s disease |
| eu00100 | [x]dementia in alzheimer's disease with late onset | Alzheimer’s disease |
| eu00111 | [x]alzheimer's disease type 1 | Alzheimer’s disease |
| eu00112 | [x]senile dementia,alzheimer's type | Alzheimer’s disease |
| eu00113 | [x]primary degen dementia of alzheimer's type, senile onset | Alzheimer’s disease |
| eu00z00 | [x]dementia in alzheimer's disease, unspecified | Alzheimer’s disease |
| eu00z11 | [x]alzheimer's dementia unspec | Alzheimer’s disease |
| f110.00 | alzheimer's disease | Alzheimer’s disease |
| f110000 | alzheimer's disease with early onset | Alzheimer’s disease |
| f110100 | alzheimer's disease with late onset | Alzheimer’s disease |
| fyu3000 | [x]other alzheimer's disease | Alzheimer’s disease |
| e004.00 | arteriosclerotic dementia | Vascular dementia |
| e004.11 | multi infarct dementia | Vascular dementia |
| e004000 | uncomplicated arteriosclerotic dementia | Vascular dementia |
| e004100 | arteriosclerotic dementia with delirium | Vascular dementia |
| e004200 | arteriosclerotic dementia with paranoia | Vascular dementia |
| e004300 | arteriosclerotic dementia with depression | Vascular dementia |
| e004z00 | arteriosclerotic dementia nos | Vascular dementia |
| eu01.00 | [x]vascular dementia | Vascular dementia |
| eu01.11 | [x]arteriosclerotic dementia | Vascular dementia |
| eu01000 | [x]vascular dementia of acute onset | Vascular dementia |
| eu01100 | [x]multi-infarct dementia | Vascular dementia |
| eu01200 | [x]subcortical vascular dementia | Vascular dementia |
| eu01300 | [x]mixed cortical and subcortical vascular dementia | Vascular dementia |
| eu01y00 | [x]other vascular dementia | Vascular dementia |
| eu01z00 | [x]vascular dementia, unspecified | Vascular dementia |
| eu02500 | [x]lewy body dementia | dementia with Lewy bodies |
| f116.00 | lewy body disease | dementia with Lewy bodies |
| eu00200 | [x]dementia in alzheimer's dis, atypical or mixed type | Mixed dementia |
| e00..11 | senile dementia | Unknown |
| e00..12 | senile/presenile dementia | Unknown |
| e000.00 | uncomplicated senile dementia | Unknown |
| e001.00 | presenile dementia | Unknown |
| e001000 | uncomplicated presenile dementia | Unknown |
| e001100 | presenile dementia with delirium | Unknown |
| e001200 | presenile dementia with paranoia | Unknown |
| e001300 | presenile dementia with depression | Unknown |
| e001z00 | presenile dementia nos | Unknown |
| e002.00 | senile dementia with depressive or paranoid features | Unknown |
| e002000 | senile dementia with paranoia | Unknown |
| e002100 | senile dementia with depression | Unknown |
| e002z00 | senile dementia with depressive or paranoid features nos | Unknown |
| e003.00 | senile dementia with delirium | Unknown |
| e012.00 | other alcoholic dementia | Unknown |
| e012.11 | alcoholic dementia nos | Unknown |
| e02y100 | drug-induced dementia | Unknown |
| e041.00 | dementia in conditions ec | Unknown |
| eu01111 | [x]predominantly cortical dementia | Unknown |
| eu02.00 | [x]dementia in other diseases classified elsewhere | Unknown |
| eu02000 | [x]dementia in pick's disease | Unknown |
| eu02100 | [x]dementia in creutzfeldt-jakob disease | Unknown |
| eu02200 | [x]dementia in huntington's disease | Unknown |
| eu02300 | [x]dementia in parkinson's disease | Unknown |
| eu02400 | [x]dementia in human immunodef virus [hiv] disease | Unknown |
| eu02y00 | [x]dementia in other specified diseases classif elsewhere | Unknown |
| eu02z00 | [x] unspecified dementia | Unknown |
| eu02z11 | [x] presenile dementia nos | Unknown |
| eu02z13 | [x] primary degenerative dementia nos | Unknown |
| eu02z14 | [x] senile dementia nos | Unknown |
| eu02z16 | [x] senile dementia, depressed or paranoid type | Unknown |
| eu04100 | [x]delirium superimposed on dementia | Unknown |
| eu10711 | [x]alcoholic dementia nos | Unknown |
| f111.00 | pick's disease | Unknown |
